# HIF‐1*α* binding to AEG‐1 promoter induced upregulated AEG‐1 expression associated with metastasis in ovarian cancer

**DOI:** 10.1002/cam4.1053

**Published:** 2017-04-12

**Authors:** Ting Zhao, Chenyan Zhao, Yanting Zhou, Jing Zheng, Shujun Gao, Yuan Lu

**Affiliations:** ^1^ Department of GynecologyObstetrics and Gynecology Hospital of Fudan UniversityShanghaiChina; ^2^ Department of PathologyObstetrics and Gynecology Hospital of Fudan UniversityShanghaiChina; ^3^ School of PharmacyEast China University of Science and TechnologyShanghaiChina; ^4^ The Diagnosis and Treatment Center of Cervical DiseaseObstetrics and Gynecology Hospital of Fudan UniversityShanghaiChina

**Keywords:** AEG‐1, HIF‐1*α*, metastasis, ovarian cancer, OVCAR3 cells

## Abstract

Ovarian cancer with the highest mortality rate among gynecological malignancies is one of common cancers among female cancer patients. As reported in recent years, AEG‐1 was associated with the occurrence, development, and metastasis of ovarian cancer, but the mechanisms remain unclear. In the current study, invasion capabilities of ovarian cancer OVCAR3 cells were measured by viral infection and transwell assay. Western blot analysis was used to evaluate the expression levels of *β*‐catenin, E‐cadherin, MMP2, and MMP9. With qRT‐PCR analysis, AEG‐1 and HIF‐1*α* gene expression were detected. We used luciferase reporter gene to measure AEG‐1 promoter activity under normoxia/hypoxia in OVCAR3 cells. Our work demonstrated that AEG‐1 significantly enhanced invasion capabilities of OVCAR3 cells and the expression levels of *β*‐catenin, E‐cadherin, MMP2, and MMP9 associated with invasion capabilities of OVCAR3 cells were upregulated. Furthermore, hypoxia enhanced invasion capabilities of OVCAR3 cells and induced AEG‐1 high gene expression, which was reversed by AEG‐1 knockdown lentivirus. HIF‐1*α* expression upregulation was induced in OVCAR3 cells after hypoxia. HIF‐1*α* knockdown lentivirus induced downregulated expression of AEG‐1 and invasion capabilities of OVCAR3 cells were also inhibited. Wild‐type AEG‐1 promoter activity under hypoxic conditions was significantly higher than that AEG‐1 mutation under normoxic conditions in the absence of hypoxia response. Our results suggested that HIF‐1*α* binds to AEG‐1 promoter to upregulate its expression, which was correlated with metastasis in ovarian cancer by inducing the expression of MMP2 and MMP9 as well as inhibiting expression of E‐cadherin and *β*‐catenin.

## Introduction

Ovarian cancer is one of common malignancy of the female genital tract worldwide. The incidence rate of ovarian cancer among female cancer patients was about 4% and ranked third, being only second to cervical cancer and endometrial cancer. Clinical ovarian cancer mortality ranks first in all gynecological tumors. The 5‐year relative survival rate for ovarian cancer was only 30–40% 1, 2. This is in part due to the metastasis of ovarian cancer with most women presenting at a late stage. Although early‐stage ovarian cancer can be successfully treated, the disease is commonly detected at advanced stages with extensive local and systemic spread and poor survival. Therefore, the discovery of novel therapeutic targets is important in the battle against ovarian cancer.

Current biomarker candidates have had insufficient performance to improve early detection efforts 3, 4, 5, and early detection strategies have not been shown to reduce mortality 6, 7. Recent genomic analyses of many human cancers have revealed that a significant number of tumors have alterations in a few core pathways 8, identifying and characterizing these core pathways provides a foundation for diagnostic and therapeutic development.

Astrocyte‐elevated gene‐1 (AEG‐1) was discovered as a novel protein induced by human immunodeficiency virus‐1 or tumor necrosis factor‐*α* in primary human fetal astrocytes 9, 10, 11. Furthermore, AEG‐1 was found as a multifunctional oncoprotein. Upregulation of AEG‐1, which was associated with the occurrence, development, and metastasis of several cancers 12, 13, 14, 15, was recognized in breast cancer, glioma, and prostate cancer 16, 17, 18. AEG‐1 was overexpressed in tumor and almost not expressed in normal tissues 19, 20. As a downstream target of Ha‐Ras, AEG‐1 played an essential role in promoting tumorigenesis, invasion, metastasis, and angiogenesis 21. Serum starvation‐induced cell death was blocked by AEG‐1 overexpression and this inhibition was mediated through PI3K‐Akt signaling pathways 16, 21, 22, 23. Overexpression of AEG‐1 promoted tumorigenesis and progression by activating ERK, Akt, and p38 MAPK pathways in hepatocellular carcinoma 24. AEG‐1 can regulate cancer invasion through upregulation of matrix metalloproteinase‐9 (MMP9), matrix metalloproteinase‐2 (MMP2), and activation of NF‐*κ*B signaling pathway 13, 16, 25, 26, 27. Most solid tumors developed regions of hypoxia as they grew and outstrip their blood supply. Hypoxia‐inducible factor 1*α* (HIF‐1*α*) is the master regulator of cell response to hypoxia since it leads to the expression of several genes involved in adaptation to decreased oxygen availability 28. Regulation of HIF‐1*α* in cells occurred as a result of which cells could adapt to hypoxic environment.

As reported recently, AEG‐1 expression increased in ovarian cancer and was associated with metastasis 29, however, the exact mechanism is still unclear. In our study, hypoxia response element (HRE) was found in AEG‐1 promoter region which could be bound to HIF‐1*α* 30. Therefore, we are intended to explore the relationship between AEG‐1 and HIF‐1*α* involved in metastasis of ovarian cancer in the current study.

## Materials and Methods

### Cell culture

Ovarian carcinoma cell line (OVCAR3) was purchased from the Cell Bank of Chinese Academy of Science (Shanghai, China). The cells were cultured in RPMI 1640 medium supplemented with 10% fetal bovine serum both from Gibco‐Invitrogen (Grand Island, NY) and 1% penicillin and streptomycin (Sigma, St. Louis, MO). Cells were maintained at 37°C in a humidified atmosphere consisting of either 5% CO_2_ and 95% air or 1% CO_2_ and 99% air.

### Lentivirus and lentivirus infection

AEG‐1 and HIF‐1*α* overexpression lentivirus and knockdown lentivirus were purchased from Shanghai R&S Biotechnology Co., Ltd.

OVCAR3 cells were seeded into 3.5 cm dishes (1 × 10^6^ cells/dish) 1 day before lentivirus infection. The next day, lentivirus was added into dishes with a multiplicity of infection (MOI) of 4 to infect cells. The infection efficiency was detected by fluorescence microscopy analysis of GFP at 48 h after infection and the efficiency was ensured higher than 90%.

### RNA extraction and quantitative real‐time PCR

Total RNA was extracted from cell lines or frozen tissues using trizol reagent and miRNeasy mini kit (Invitrogen). The qRT‐PCR reactions were performed using SYBR Green I Master Mix (CoWin Biosciences, China) according to the manufacturer's instructions. And iQ‐5 (Bio‐Rad) was used to monitor the PCR in real‐time. Primers for E‐cadherin, *β*‐catenin, MMP‐2, MMP‐9, and GAPDH were designed using Primer Bank. The average Ct, from triplicate assays, was used for further calculations. Relative expression levels were normalized to control. The endogenous U6 snRNA was chosen as the internal control.

### Western blot analysis

Total proteins of cells were extracted according to the protocol of protein extraction kit (KeyGen, China), and the protein concentration of each sample was determined using the bicinchoninic acid protein assay kit (Pierce Biotechnology, Rockford, IL). Equivalent quantities of protein were separated by 12% SDS polyacrylamide gels and transferred to nitrocellulose membranes. Membranes were blocked with 10% defatted milk (10% BSA for p‐protein) and then incubated with the appropriate primary antibody overnight at 4°C. They were next washed and incubated with the corresponding horseradish peroxidase (HRP)‐conjugated secondary antibody for 1 h. Bound secondary antibody was visualized using an enhanced chemiluminescence (ECL) system (Pierce Biotechnology). The primary antibodies used were anti‐GAPDH (1:1000, Cell Signal Technology, Danvers, MA), anti‐AEG‐1 (1:10000, Abcam, USA), anti‐E‐cadherin(1:200 Boster bio., Shanghai, China), anti‐*β*‐catenin (1:1000, Cell Signal Technology), anti‐MMP2 (1:1000, Abcam, USA), and anti‐MMP9 (1:1000, Cell Signal Technology). The results were normalized to GAPDH to correct for loading.

### Invasion assay in vitro

Transwell invasion experiments were performed with 24‐well matrigel‐coated chambers from BD Biosciences (Bedford, MA). OVCAR3 cells infected with lentivirus were seeded into the upper chambers at the density of 5 × 10^4^ cells in 200 *μ*L serum‐free RPMI 1640 medium. The lower chambers were filled with 500 *μ*L RPMI 1640 medium containing 10% FBS. After 24 h of incubation, noninvading cells on the top of the membrane were removed by scraping. Invaded cells on the bottom of the membrane were fixed with 100% methanol for 2 min, followed by staining with 0.05% crystal violet. The invaded cells on the membrane were then counted under a microscope. The invaded cells were collected and lysed in 500 *μ*L lysis buffer (Bedford). Then, absorbance (OD, optical density) of invaded cell lysates was read at 570 nm. Invasion studies were repeated three times.

### Luciferase assay

OVCAR3 cells were seeded at a density of 1 × 10^5^ cells/well into 24‐well culture plates (Corning) in RPMI 1640 medium containing 10% FBS without antibiotics. After 24 h, the cells were cotransfected with AEG‐1 promoter or AEG‐1 promoter mutation in the absence of hypoxia‐response element (HRE), firefly luciferase reporter plasmid, and *Renilla* luciferase plasmid according to the manufacturer's instructions. Cells were incubated for 24 h under hypoxia condition, and using normoxia condition as a control group. Then, cell lysates were prepared and luciferase activities were measured using the dual‐luciferase reporter assay system (Promega, Madison, WI). Firefly luciferase activity was normalized to the activity of *Renilla* luciferase.

### Data analysis

Results are presented as mean ± standard deviation (SD) of three samples. Significant differences in the mean values were evaluated by Student's unpaired *t*‐test. Places needing multiple comparisons were evaluated by one‐way ANOVA with Bonferroni correction. *P*‐value of 0.05 or less was considered to be statistically significant.

## Results

### AEG‐1 enhanced invasion capability of OVCAR3 cells

**Figure 1:**
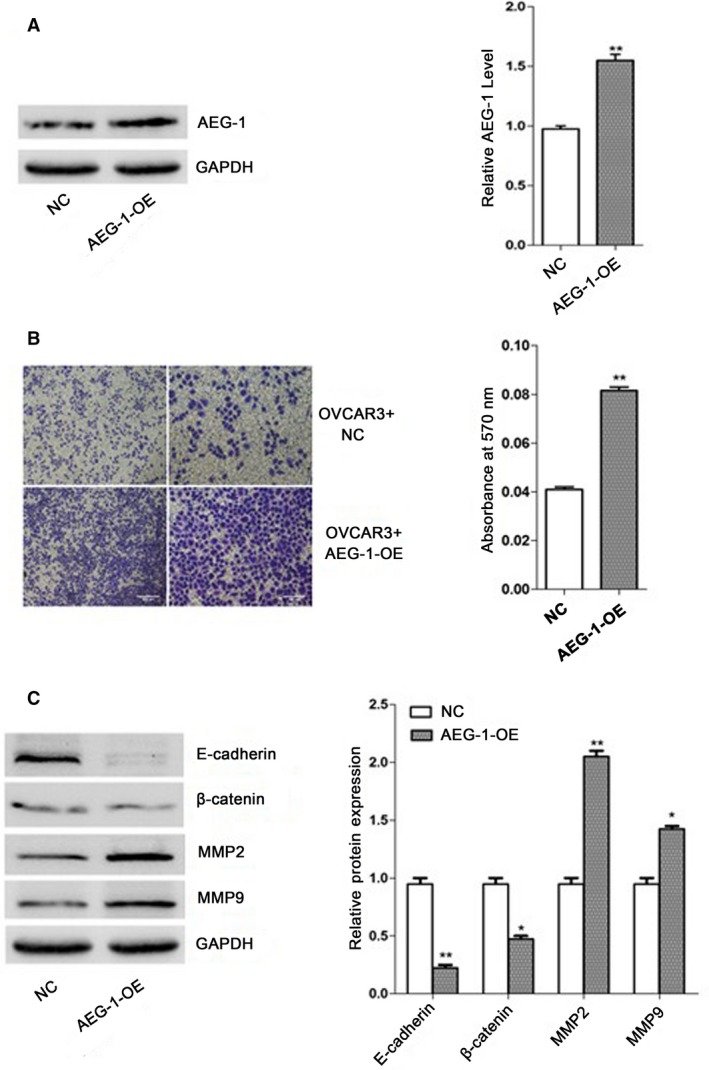
AEG‐1 enhanced the capability of OVCAR3 cells invasion. After infection with AEG‐1 over expression /negative control lentivirus for 72h, OVCAR3 cells were collected for Transwell assay. Cell lysates were then subjected to Western blotting to measure the protein expression of AEG‐1, E‐cadherin, β‐catenin, MMP2, and MMP9. The results were normalized to GAPDH to correct for loading. Data given are mean ± SD, *n* = 3. *P <0.05, **P <0.01 versus negative control. (A) AEG‐1 expression, (B) Invasion capability and absorbances [optical density (OD)] of invaded cell lysates read at 570nm, (C) MMP2, MMP9, E‐cadherin and β‐catenin protein expressions of OVCAR3 cells infected with over expression /negative control lentivirus.

**Figure 2:**
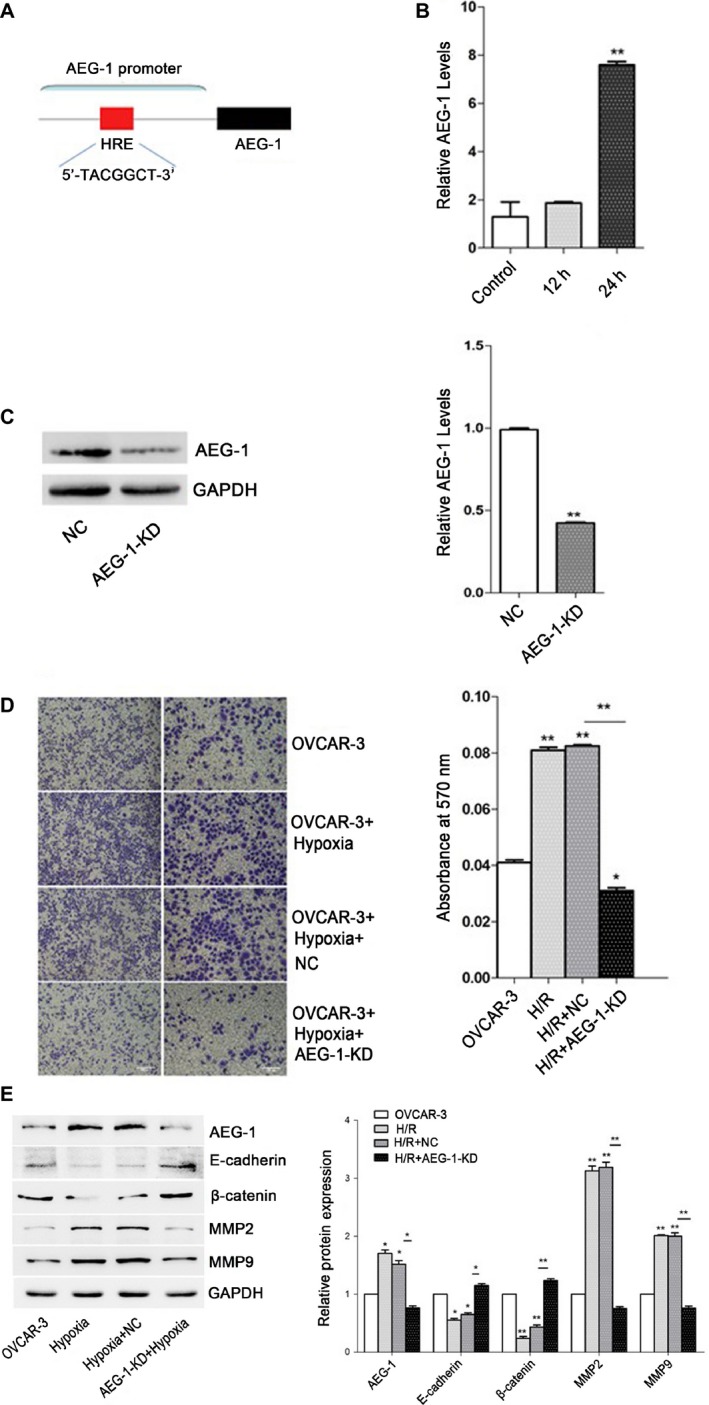
Invasion capability of OVCAR3 cells increased after hypoxia. OVCAR3 cells were maintained in a humidified atmosphere consisting of 1% CO2 and 99% air for 12h and 24h. Then the cells were collected for qPCR analysis, Western blotting and transwell assay. Data given are mean ± SD, *n* = 3. (A) Schematic representation of AEG‐1 promoter. (B) The expression of AEG‐1 in OVCAR3 cells after hypoxia and control analyzed by qPCR. ***P <0.001 versus control. (C) The expression of AEG‐1 in OVCAR3 cells after infection with AEG‐1 knock down/negative control lentivirus analyzed by Western blotting. The results were normalized to GAPDH to correct for loading. **P <0.01 versus negative control. (D) Invasion capability of control and OVCAR3 cells after hypoxia, followed by infection with AEG‐1 knock down/negative control lentivirus. The absorbances [optical density (OD)] of invaded cell lysates were read at 570nm. *P <0.05, **P <0.01 versus control. ***P <0.001, OVCAR3 cells after hypoxia and infection with AEG‐1 knockdown lentivirus versus negative control. (E) Protein expressions of AEG‐1, MMP2, MMP9, E‐cadherin and β‐catenin analyzed by Western blotting. The results were normalized to GAPDH to correct for loading. *P <0.05, **P <0.01 versus control.

To investigate the effect of AEG‐1 upregulation invasion of OVCAR3 cells, we performed transwell assay and measured the levels of E‐cadherin, *β*‐catenin, MMP2, and MMP9 associated with cancer invasion and metastasis 30, 31. Expression of AEG‐1 in OVCAR3 cells infected with AEG‐1 overexpression lentivirus was significantly increased (Fig. 1A). Figure 2B showed the changes of invasion capacity of OVCAR3 cells. OVCAR3 cells infected with AEG‐1 overexpression lentivirus showed significantly increased invasion capability compared with negative control lentivirus infected‐OVCAR3 cells (*P* < 0.01). As shown in Figure 1C, the protein expressions of MMP2 and MMP9, the two important molecules involved in tumor invasion 31, 32 in AEG‐1 over expression group, were significantly increased, while expressions of E‐cadherin and *β*‐catenin, important regulatory markers for epithelial–mesenchymal transition (EMT) recognized crucial event in invasion and metastasis 32, considerably decreased compared with negative control group.

### Hypoxia affected the expression of AEG‐1 and stimulated invasion of OVCAR3 cells

Computer‐assisted analysis of biological sequences indicated that HRE consensus (5′‐TACGTGCT‐3′) exists in the AEG‐1 promoter (Fig. 2A). To confirm the upregulating effects of hypoxia, we performed qPCR analysis, western blotting, and transwell assay. Expressions of AEG‐1 in OVCAR3 cells after hypoxia for 24 h were significantly increased (**P < 0.01), which was in a time‐dependent manner (Fig. 2B). Expressions of AEG‐1 in OVCAR3 cells infected with AEG‐1 knockdown lentivirus after hypoxia for 24 h were significantly decreased (*P < 0.05) (Fig. 2C). As shown in Figure 2D, invasion capability of OVCAR3 cells after hypoxia for 24 h was significantly upregulated (**P < 0.01) compared with that of OVCAR3 cells without hypoxia. However, invasion capability of OVCAR3 cells infected with AEG‐1 knockdown lentivirus significantly decreased (*P < 0.05). The protein expressions of MMP2 and MMP9 in AEG‐1 knockdown group were significantly decreased, as well as expressions of E‐cadherin and *β*‐catenin increased compared with negative control group (Fig. 2E). However, no significant difference in protein expressions was found between OVCAR3 cells and AEG‐1 knockdown ones.

### HIF‐1α induced expression of AEG‐1

**Figure 3:**
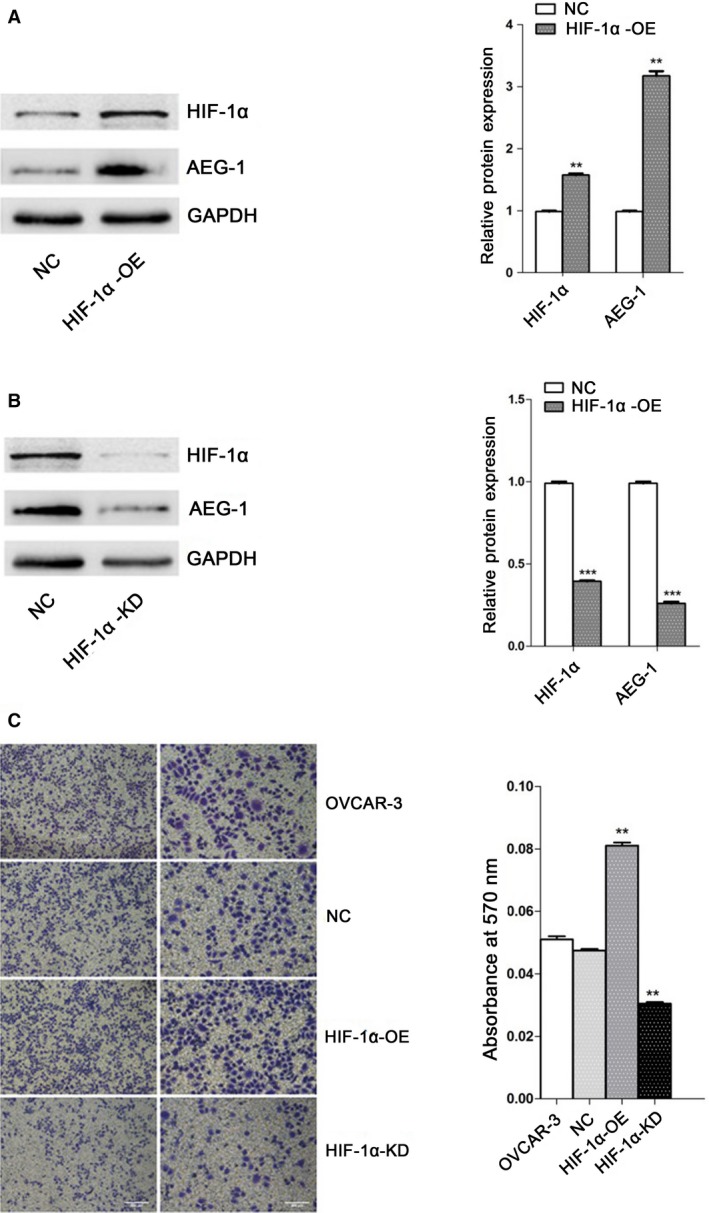
HIF‐1α induced up‐regulated expression of AEG‐1 and invasion capability of OVCAR3 cells. OVCAR3 cells were infected with HIF‐1αoverexpression/knockdown lentivirus for 72h. Then the cells were collected for transwell assay. Cell lysates were subjected to Western blotting analysis. The results were normalized to GAPDH to correct for loading. Data given are mean ± SD, *n* = 3. (A) The expression of AEG‐1 and HIF‐1α in OVCAR3 cells infected with HIF‐1α overexpression/ negative control lentivirus. **P <0.01 versus negative control. (B) The expression of AEG‐1 and HIF‐1α in OVCAR3 cells infected with HIF‐1α knockdown/negative control lentivirus. ***P <0.001 versus negative control. (C) Invasion capability of control and OVCAR3 cells after infection with HIF‐1α overexpression/knockdown and negative control lentivirus. The absorbances [optical density (OD)] of invaded cell lysates were read at 570nm. **P <0.01 versus control.

To investigate the correlation between HIF‐1*α* and expression of AEG‐1, we comparatively analyzed AEG‐1 protein expression profiles in OVCAR3 cells infected with HIF‐1*α* overexpression/knockdown lentivirus with different metastatic ability. Western blot analysis revealed that AEG‐1 expression was upregulated in OVCAR3 cells infected with HIF‐1*α* overexpression lentivirus (Fig. 3A), which was reversed by knockdown HIF‐1*α* (Fig. 3B). The results of transwell assay showed that overexpression of HIF‐1*α* promoted metastasis in OVCAR3 cells. Invasion capability of OVCAR3 cells infected with HIF‐1*α* knockdown lentivirus was significantly decreased compared with normal/negative control OVCAR3 cells (*^*^ *P* < 0.01) (Fig. 3C).

### HIF‐1α regulates AEG‐1 promoter activity

**Figure 4:**
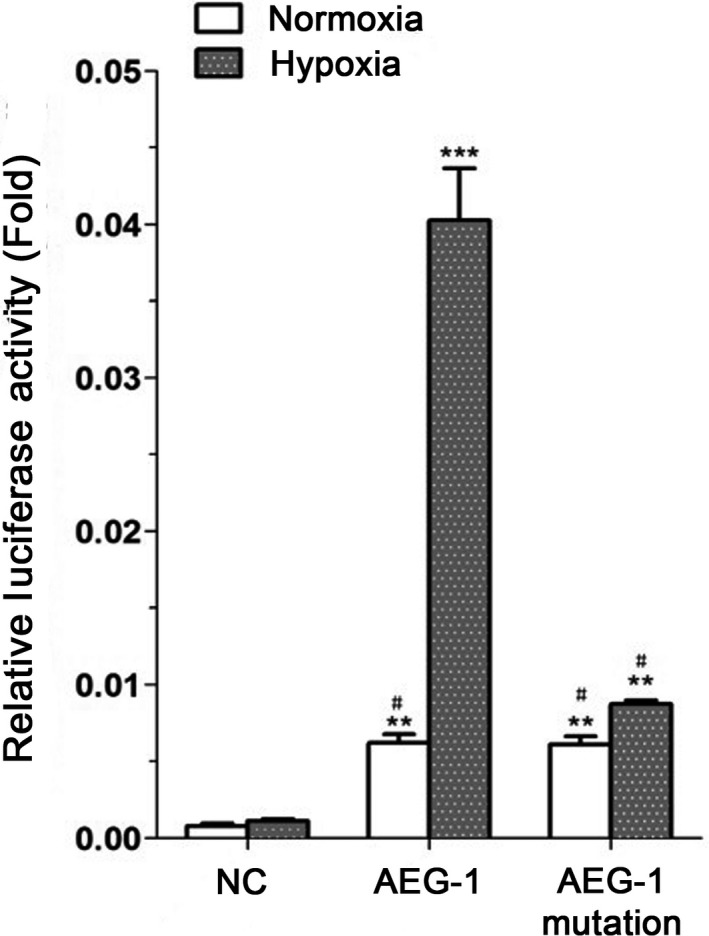
Hypoxia induced up‐regulated AEG‐1 promoter activity via HIF‐1α. AEG‐1 luciferase construct was cotransfected in OVCAR3 cells. Cells were incubated under hypoxic/normoxic conditions for 24 hours. Luciferase activities of cell lysates were measured. Firefly luciferase activity was normalized to the activity of Renilla luciferase. Data given are mean ± SD, *n* = 3. **P <0.01, means versus negative control. ***P <0.001, means wild‐type AEG‐1 group under hypoxia versus normoxia group. # means there was no significant difference among AEG‐1 mutation group under hypoxia, wild‐type AEG‐1 and AEG‐mutation group under normoxia.

To further understand the relevance of HIF‐1*α* and expression of AEG‐1, we used luciferase reporter gene to measure AEG‐1 promoter activity under normoxic/hypoxic conditions in OVCAR3 cells. Wild‐type AEG‐1 promoter activity under hypoxic conditions was 6.79 times higher than under normoxic conditions, which was consistent with HIF‐1*α*. Promoter activity of AEG‐1 mutation in the absence of HRE under hypoxia was significantly decreased compared with wild‐type AEG‐1. Furthermore, there was no significant difference among AEG‐1 mutation group under hypoxia, wild‐type AEG‐1 under normoxia, and AEG mutation under normoxia, suggesting that hypoxia contributes to AEG‐1 promoter activity via HIF‐1*α* upregulated expression (Fig. 4).

## Discussion

AEG‐1 has been increasingly recognized as an oncogene and was involved in many aspects of tumorigenesis, including enhanced anchorage‐independent growth, migration, angiogenesis, and protection from serum starvation‐induced apoptosis 16, 23. The role of AEG‐1 in diverse cancers has been extensively studied with regard to be proangiogenic both in vitro and in vivo and can also augment expression of key angiogenesis molecules, such as angiopoietin‐1 (Ang1) and MMP‐2 23. A previous study reported higher AEG‐1 expression in ovarian cancer than in normal ovarian tissue 33. Here, we propose a mechanism of metastatic progression, in which AEG‐1 level is a key regulator of ovarian cancer metastasis. In our study, we performed the infection of OVCAR3 cells with lentivirus and obtained the AEG‐1 overexpressed ovarian cells. Quantitatively, the protein level of AEG‐1 in AEG‐1 overexpressed OVCAR3 cells is higher than negative control lentivirus‐infected OVCAR3 cells (Fig. 1A). OVCAR3 cells infected with AEG‐1 overexpression lentivirus showed significantly increased invasion capability compared with negative control group (Fig. 1B). This is consistent with our proposed mechanism in which overexpression of AEG‐1 in OVCAR3 cells leads to enhanced invasion ability of ovarian cancer. However, other possible tumor metastatic promoting molecules (MMP‐2, MMP‐9) and repressors (E‐cadherin, *β*‐catenin) could also be involved in the regulation of ovarian cancer metastasis.

Epithelial–mesenchymal transformation (EMT), recently proved to be involved in tumor invasion and metastasis, is a critical step during embryonic development during which epithelial cells lose the cell adhesion, molecule E‐cadherin, and convert into migratory cells 34, 35, 36. MMP‐2 and MMP‐9 are upregulated in human ovarian carcinomas. They promote invasion, metastasis, growth, and the survival of malignant cells 37, 38, 39. *β*‐catenin is a main downstream effector of the canonical Wnt signaling pathway, which has a dual role in EMT 32. Furthermore, loss of E‐cadherin associated with enhanced cell invasion and metastasis has been observed in ovarian cancer during EMT 40, 41, 42. Analysis of protein level revealed that MMP‐2 and MMP‐9 in AEG‐1 overexpressed OVCAR3 cells was upregulated leading to higher invasion of OVCAR3 cells (Fig. 1C). In contrast, E‐cadherin and *β*‐catenin were highly downregulated in AEG‐1 overexpressed ovarian cancer cells (Fig. 1C), thereby leading both to the loss of cell–cell adhesion and to the increased ovarian cancer invasion (Fig. 1B).

The microenvironment of solid cancerous masses closely linked to cell proliferation, invasion, and metastasis often characterized by low oxygen and pH and insufficient nutrients because of inadequate circulation 43. The transcription factor HIF‐1*α* is a critical mediator of the hypoxic response and upregulates expression of proteins that promote angiogenesis, anaerobic metabolism, and many other survival pathways 44. HIF‐1*α* is a key cellular survival protein under hypoxia and is associated with tumor progression and metastasis in various solid tumors. HIF‐1*α* expression allows metabolic adaptation to low oxygen availability. Inspired by these lines of evidences, we plan to test whether hypoxia, another process associated with tumorigenesis and metastasis of ovarian cancers, is functionally involved in expression of AEG‐1. Interestingly, we found that hypoxia could significantly induce AEG‐1 expression upregulation in ovarian cancer cells in a time‐dependent manner (Fig. 2B). To further confirm that ovarian cancer metastasis is mediated by AEG‐1, we generated an AEG‐1 knockdown OVCAR3 cells under hypoxia. We observed the protein level of AEG‐1 of AEG‐1 knockdown OVCAR3 cells is notably lower than the negative control one (Fig. 2C). And then we utilized the transwell migration assay to visualize invasion of AEG‐1 knockdown OVCAR3 cells after hypoxia treatment. Our data demonstrated that invasion capability of OVCAR3 cells infected with AEG‐1 knockdown lentivirus significantly decreased compared with the negative control one (Fig. 2D). In this experiment, AEG‐1 induction in ovarian cancer likely occurs through coordinated control by hypoxia‐regulated gene expression, and as previously reported, AEG‐1 was recently shown to induce HIF‐1*α* as well as Tie2 and VEGF 23, which forms a positive feedback loop. This is consistent with other reports that AEG‐1 expression may allow survival and continued cell growth in the midst of diminishing oxygen and energy supplies, processes which would render cells without induced AEG‐1 expression more susceptible to necrosis 45.

With assistance of computer analysis of biological sequences, HRE was found in AEG‐1 promoter region (Fig. 2A). Therefore, we proposed that HIF‐1*α* would bind to HRE in the promoters of AEG‐1 after hypoxia treatment and enhance AEG‐1 promoter activity to induce tumor growth and migration. Our findings showed that HIF‐1*α* activity is crucial for AEG‐1 induction during the period of hypoxia. Overexpression of HIF‐1*α* upregulated AEG‐1 expression and enhanced migration of ovarian cancer cells (Fig. 3A and C) by inducing the expression of MMP2 and MMP9 as well as inhibiting the expression of E‐cadherin and *β*‐catenin. Consistently, when AEG‐1 was knocked down in OVCAR3 cells, we observed a significant decrease in the protein level of AEG‐1 (Fig. 3B and C). In addition, the results of luciferase reporter gene assay (Fig. 4) confirmed that HIF‐1*α* binds to AEG‐1 promoter and upregulated its activity under hypoxia. Therefore, AEG‐1 with enhanced migration and invasion may be due to activation by HIF‐1*α*.

About 70% patients affected by epithelial ovarian carcinomas were detected at advanced stages; the lesions of these patients implant on the peritoneum all over pelvic and peritoneal cavity, especially the omentum; this feature of ovarian carcinoma leads to great difficulty in achieving the goal of optimal debulking surgery, which is one of the most important factors affecting the survivals. Even if cytoreductive surgery and platinum‐based chemotherapy were implemented, the patients may still suffer from recurrence and spreading of the resurgent lesions. In these processes, the mechanism of metastasis remains unclear. Our studies have shown that AEG‐1 is closely correlated with EMT and plays an important role in the migration and invasion of ovarian cancer cells in vitro. HIF‐1*α* binds to AEG‐1 promoter and induces upregulated AEG‐1 expression to enhance metastasis in ovarian cancer by increasing MMP2 and MMP9 expression as well as attenuating E‐cadherin and *β*‐catenin expression during hypoxia, which is a typical feature of tumor microenvironment. This pathway of AEG‐1 induction may be a pivotal mediator of ovarian cancer and could have important implication for a potential therapeutic target as well.

In summary, our studies showed a novel pathway of HIF‐1*α* associated AEG‐1 induction, which is a protein that mediates the migration and invasion of ovarian cancer cells. These findings at least partially reveal the mechanism of metastasis in ovarian cancer and may facilitate the development of novel molecular strategies for the treatment of this tumor.

## Conflict of Interest

None declared.

## References

[1] Jemal, A. , F. Bray , M. M. Center , J. Ferlay , E. Ward , and D. Forman . 2011 Global cancer statistics. CA Cancer J. Clin. 61:69–90. 21296855 10.3322/caac.20107

[2] Karim‐Kos, H. E. , E. de Vries , I. Soerjomataram , V. Lemmens , S. Siesling , and J. W. Coebergh . 2008 Recent trends of cancer in Europe: a combined approach of incidence, survival and mortality for 17 cancer sites since the 1990s. Eur. J. Cancer 44:1345–1389. 18280139 10.1016/j.ejca.2007.12.015

[3] Zhu, C. S. , P. F. Pinsky , D. W. Cramer , D. F. Ransohoff , P. Hartge , R. M. Pfeiffer , et al. 2011 A framework for evaluating biomarkers for early detection: validation of biomarker panels for ovarian cancer. Cancer Prev. Res. (Phila.) 4:375–383. 21372037 10.1158/1940-6207.CAPR-10-0193 PMC3057372

[4] Cramer, D. W. , R. C. Jr. Bast , C. D. Berg , E. P. Diamandis , A. K. Godwin , P. Hartge , et al. 2011 Ovarian cancer biomarker performance in prostate, lung, colorectal, and ovarian cancer screening trial specimens. Cancer Prev. Res. (Phila.) 4:365–374. 21372036 10.1158/1940-6207.CAPR-10-0195 PMC3085251

[5] Trabert, B. , R. B. Ness , W. H. Lo‐Ciganic , M. A. Murphy , E. L. Goode , E. M. Poole , et al. 2014 Aspirin, nonaspirin nonsteroidal anti‐inflammatory drug, and acetaminophen use and risk of invasive epithelial ovarian cancer: a pooled analysis in the Ovarian Cancer Association Consortium. J. Natl Cancer Inst. 106: djt431. 24503200 10.1093/jnci/djt431 PMC3924755

[6] Buys, S. S. , E. Partridge , M. H. Greene , P. C. Prorok , D. Reding , T. L. Riley , et al. 2005 Ovarian cancer screening in the prostate, lung, colorectal and ovarian (PLCO) cancer screening trial: findings from the initial screen of a randomized trial. Am. J. Obstet. Gynecol. 193:1630–1639. 16260202 10.1016/j.ajog.2005.05.005

[7] Buys, S. S. , E. Partridge , A. Black , C. C. Johnson , L. Lamerato , C. Isaacs , et al. 2011 Effect of screening on ovarian cancer mortality: the Prostate, Lung, Colorectal and Ovarian (PLCO) Cancer Screening Randomized Controlled Trial. JAMA 305:2295–2303. 21642681 10.1001/jama.2011.766

[8] Rodriguez, N. , J. Yang , K. Hasselblatt , S. Liu , Y. Zhou , J. A. Rauh‐Hain , et al. 2012 Casein kinase I epsilon interacts with mitochondrial proteins for the growth and survival of human ovarian cancer cells. EMBO Mol. Med. 4:952–963. 22707389 10.1002/emmm.201101094 PMC3491827

[9] Su, Z. Z. , D. C. Kang , Y. Chen , O. Pekarskaya , W. Chao , D. J. Volsky , et al. 2003 Identification of gene products suppressed by human immunodeficiency virus type 1 infection or gp120 exposure of primary human astrocytes by rapid subtraction hybridization. J. Neurovirol. 9:372–389. 12775420 10.1080/13550280390201263

[10] Kang, D. C. , Z. Z. Su , D. Sarkar , L. Emdad , D. J. Volsky , and P. B. Fisher . 2005 Cloning and characterization of HIV‐1‐inducible astrocyte elevated gene‐1, AEG‐1. Gene 353:8–15. 15927426 10.1016/j.gene.2005.04.006

[11] Su, Z. Z. , D. C. Kang , Y. Chen , O. Pekarskaya , W. Chao , D. J. Volsky , et al. 2002 Identification and cloning of human astrocyte genes displaying elevated expression after infection with HIV‐1 or exposure to HIV‐1 envelope glycoprotein by rapid subtraction hybridization, RaSH. Oncogene 21:3592–3602. 12032861 10.1038/sj.onc.1205445

[12] Brown, D. M. , and E. Ruoslahti . 2004 Metadherin, a cell surface protein in breast tumors that mediates lung metastasis. Cancer Cell 5:365–374. 15093543 10.1016/s1535-6108(04)00079-0

[13] Emdad, L. , D. Sarkar , Z. Z. Su , A. Randolph , H. Boukerche , K. Valerie , et al. 2006 Activation of the nuclear factor kappaB pathway by astrocyte elevated gene‐1: implications for tumor progression and metastasis. Cancer Res. 66:1509–1516. 16452207 10.1158/0008-5472.CAN-05-3029

[14] Hu, G. , R. A. Chong , Q. Yang , Y. Wei , M. A. Blanco , F. Li , et al. 2009 MTDH activation by 8q22 genomic gain promotes chemoresistance and metastasis of poor‐prognosis breast cancer. Cancer Cell 15:9–20. 19111877 10.1016/j.ccr.2008.11.013 PMC2676231

[15] Liu, L. , J. Wu , Z. Ying , B. Chen , A. Han , Y. Liang , et al. 2010 Astrocyte elevated gene‐1 upregulates matrix metalloproteinase‐9 and induces human glioma invasion. Cancer Res. 70:3750–3759. 20388776 10.1158/0008-5472.CAN-09-3838

[16] Emdad, L. , D. Sarkar , Z. Z. Su , S. G. Lee , D. C. Kang , J. N. Bruce , et al. 2007 Astrocyte elevated gene‐1: recent insights into a novel gene involved in tumor progression, metastasis and neurodegeneration. Pharmacol. Ther. 114:155–170. 17397930 10.1016/j.pharmthera.2007.01.010 PMC2039930

[17] Li, J. , N. Zhang , L. B. Song , W. T. Liao , L. L. Jiang , L. Y. Gong , et al. 2008 Astrocyte elevated gene‐1 is a novel prognostic marker for breast cancer progression and overall patient survival. Clin. Cancer Res. 14:3319–3326. 18519759 10.1158/1078-0432.CCR-07-4054

[18] Kikuno, N. , H. Shiina , S. Urakami , K. Kawamoto , H. Hirata , Y. Tanaka , et al. 2007 Knockdown of astrocyte‐elevated gene‐1 inhibits prostate cancer progression through upregulation of FOXO3a activity. Oncogene 26:7647–7655. 17563745 10.1038/sj.onc.1210572

[19] Yoo, B. K. , L. Emdad , Z. Z. Su , A. Villanueva , D. Y. Chiang , N. D. Mukhopadhyay , et al. 2009 Astrocyte elevated gene‐1 regulates hepatocellular carcinoma development and progression. J. Clin. Invest. 119:465–477. 19221438 10.1172/JCI36460 PMC2648696

[20] Milhem, M. M. , T. Knutson , S. Yang , D. Zhu , X. Wang , K. K. Leslie , et al. 2011 Correlation of MTDH/AEG‐1 and HOTAIR expression with metastasis and response to treatment in sarcoma patients. J. Cancer Sci. Ther. S5:000. PMC3612017 23543869

[21] Lee, S. G. , Z. Z. Su , L. Emdad , D. Sarkar , and P. B. Fisher . 2006 Astrocyte elevated gene‐1 (AEG‐1) is a target gene of oncogenic Ha‐ras requiring phosphatidylinositol 3‐kinase and c‐Myc. Proc. Natl Acad. Sci. USA 103:17390–17395. 17088530 10.1073/pnas.0608386103 PMC1859939

[22] Lee, S. G. , Z. Z. Su , L. Emdad , D. Sarkar , T. F. Franke , and P. B. Fisher . 2008 Astrocyte elevated gene‐1 activates cell survival pathways through PI3K‐Akt signaling. Oncogene 27:1114–1121. 17704808 10.1038/sj.onc.1210713

[23] Emdad, L. , S. G. Lee , Z. Z. Su , H. Y. Jeon , H. Boukerche , D. Sarkar , et al. 2009 Astrocyte elevated gene‐1 (AEG‐1) functions as an oncogene and regulates angiogenesis. Proc. Natl Acad. Sci. USA 106:21300–21305. 19940250 10.1073/pnas.0910936106 PMC2795510

[24] Srivastava, J. , A. Siddiq , L. Emdad , P. K. Santhekadur , D. Chen , R. Gredler , et al. 2012 Astrocyte elevated gene‐1 promotes hepatocarcinogenesis: novel insights from a mouse model. Hepatology 56:1782–1791. 22689379 10.1002/hep.25868 PMC3449036

[25] Yu, C. , K. Chen , H. Zheng , X. Guo , W. Jia , M. Li , et al. 2009 Overexpression of astrocyte elevated gene‐1 (AEG‐1) is associated with esophageal squamous cell carcinoma (ESCC) progression and pathogenesis. Carcinogenesis 30:894–901. 19304953 10.1093/carcin/bgp064

[26] Sarkar, D. , E. S. Park , L. Emdad , S. G. Lee , Z. Z. Su , and P. B. Fisher . 2008 Molecular basis of nuclear factor‐kappaB activation by astrocyte elevated gene‐1. Cancer Res. 68:1478–1484. 18316612 10.1158/0008-5472.CAN-07-6164

[27] Wang, Z. , C. J. Cao , L. L. Huang , Z. F. Ke , C. J. Luo , Z. W. Lin , et al. 2015 EFEMP1 promotes the migration and invasion of osteosarcoma via MMP‐2 with induction by AEG‐1 via NF‐kappaB signaling pathway. Oncotarget 6:14191–14208. 25987128 10.18632/oncotarget.3691 PMC4546460

[28] Semenza, G. L. 2001 Hypoxia‐inducible factor 1: oxygen homeostasis and disease pathophysiology. Trends Mol. Med. 7:345–350. 11516994 10.1016/s1471-4914(01)02090-1

[29] Zhou, B. , J. Yang , B. Shu , K. Liu , L. Xue , N. Su , et al. 2015 Overexpression of astrocyte‐elevated gene‐1 is associated with ovarian cancer development and progression. Mol. Med. Rep. 11:2981–2990. 25483832 10.3892/mmr.2014.3056

[30] Guadall, A. , M. Orriols , R. Rodriguez‐Calvo , O. Calvayrac , J. Crespo , R. Aledo , et al. 2011 Fibulin‐5 is up‐regulated by hypoxia in endothelial cells through a hypoxia‐inducible factor‐1 (HIF‐1alpha)‐dependent mechanism. J. Biol. Chem. 286:7093–7103. 21193390 10.1074/jbc.M110.162917 PMC3044966

[31] Chen, D. L. , Y. F. Ping , S. C. Yu , J. H. Chen , X. H. Yao , X. F. Jiang , et al. 2009 Downregulating FPR restrains xenograft tumors by impairing the angiogenic potential and invasive capability of malignant glioma cells. Biochem. Biophys. Res. Commun. 381:448–452. 19233142 10.1016/j.bbrc.2009.02.065

[32] Yang, L. , Y. F. Ping , X. Yu , F. Qian , Z. J. Guo , C. Qian , et al. 2011 Gastric cancer stem‐like cells possess higher capability of invasion and metastasis in association with a mesenchymal transition phenotype. Cancer Lett. 310:46–52. 21782323 10.1016/j.canlet.2011.06.003

[33] Li, C. , J. Liu , R. Lu , G. Yu , X. Wang , Y. Zhao , et al. 2011 AEG ‐1 overexpression: a novel indicator for peritoneal dissemination and lymph node metastasis in epithelial ovarian cancers. Int. J. Gynecol. Cancer 21:602–608. 21543927 10.1097/IGC.0b013e3182145561

[34] Yilmaz, M. , and G. Christofori . 2009 EMT, the cytoskeleton, and cancer cell invasion. Cancer Metastasis Rev. 28:15–33. 19169796 10.1007/s10555-008-9169-0

[35] Yang, J. , and R. A. Weinberg . 2008 Epithelial‐mesenchymal transition: at the crossroads of development and tumor metastasis. Dev. Cell 14:818–829. 18539112 10.1016/j.devcel.2008.05.009

[36] Thiery, J. P. , H. Acloque , R. Y. Huang , and M. A. Nieto . 2009 Epithelial‐mesenchymal transitions in development and disease. Cell 139:871–890. 19945376 10.1016/j.cell.2009.11.007

[37] Al‐Alem, L. , and T. E. Jr. Curry . 2015 Ovarian cancer: involvement of the matrix metalloproteinases. Reproduction 150:R55–R64. 25918438 10.1530/REP-14-0546 PMC4955511

[38] Che, Y. L. , S. J. Luo , G. Li , M. Cheng , Y. M. Gao , X. M. Li , et al. 2015 The C3G/Rap1 pathway promotes secretion of MMP‐2 and MMP‐9 and is involved in serous ovarian cancer metastasis. Cancer Lett. 359:241–249. 25617801 10.1016/j.canlet.2015.01.019

[39] Guo, F. , J. Tian , M. Cui , M. Fang , and L. Yang . 2015 Downregulation of matrix metalloproteinase 9 by small interfering RNA inhibits the tumor growth of ovarian epithelial carcinoma in vitro and in vivo. Mol. Med. Rep. 12:753–759. 25738807 10.3892/mmr.2015.3425

[40] Chen, J. , L. Wang , L. V. Matyunina , C. G. Hill , and J. F. McDonald . 2011 Overexpression of miR‐429 induces mesenchymal‐to‐epithelial transition (MET) in metastatic ovarian cancer cells. Gynecol. Oncol. 121:200–205. 21277012 10.1016/j.ygyno.2010.12.339

[41] Latifi, A. , K. Abubaker , N. Castrechini , A. C. Ward , C. Liongue , F. Dobill , et al. 2011 Cisplatin treatment of primary and metastatic epithelial ovarian carcinomas generates residual cells with mesenchymal stem cell‐like profile. J. Cell. Biochem. 112:2850–2864. 21618587 10.1002/jcb.23199

[42] Rosano, L. , R. Cianfrocca , F. Spinella , V. Di Castro , M. R. Nicotra , A. Lucidi , et al. 2011 Acquisition of chemoresistance and EMT phenotype is linked with activation of the endothelin A receptor pathway in ovarian carcinoma cells. Clin. Cancer Res. 17:2350–2360. 21220476 10.1158/1078-0432.CCR-10-2325

[43] Tamura, K. , M. Yoshie , E. Miyajima , M. Kano , and E. Tachikawa . 2013 Stathmin Regulates Hypoxia‐Inducible Factor‐1alpha Expression through the Mammalian Target of Rapamycin Pathway in Ovarian Clear Cell Adenocarcinoma. ISRN Pharmacol. 2013:279593. 23819061 10.1155/2013/279593 PMC3683482

[44] Proulx‐Bonneau, S. , and B. Annabi . 2011 The primary cilium as a biomarker in the hypoxic adaptation of bone marrow‐derived mesenchymal stromal cells: a role for the secreted frizzled‐related proteins. Biomark Insights 6:107–118. 22084569 10.4137/BMI.S8247 PMC3201088

[45] Noch, E. , M. Bookland , and K. Khalili . 2011 Astrocyte‐elevated gene‐1 (AEG‐1) induction by hypoxia and glucose deprivation in glioblastoma. Cancer Biol. Ther. 11:32–39. 21084864 10.4161/cbt.11.1.13835 PMC3047099

